# Relationships for vitamin D with childhood height growth velocity and low bone mineral density risk

**DOI:** 10.3389/fnut.2023.1081896

**Published:** 2023-02-03

**Authors:** Pei Xiao, Hong Cheng, Liange Wang, Dongqing Hou, Haibo Li, Xiaoyuan Zhao, Xianghui Xie, Jie Mi

**Affiliations:** ^1^Center for Non-Communicable Disease Management, Beijing Children's Hospital, Capital Medical University, National Center for Children's Health, Beijing, China; ^2^Department of Epidemiology, Capital Institute of Pediatrics, Beijing, China; ^3^Beijing Miyun Primary and Secondary School Health Center, Beijing, China; ^4^Child Health Big Data Research Center, Capital Institute of Pediatrics, Beijing, China; ^5^Division of Birth Cohort Study, Fujian Maternity and Child Health Hospital, Affiliated Hospital of Fujian Medical University, Fuzhou, China; ^6^Capital Institute of Pediatrics, Beijing, China

**Keywords:** vitamin D, height growth, low bone mineral density, cohort study, child

## Abstract

**Objective:**

To investigate how serum 25-hydroxyvitamin D (25[OH]D) affects height growth velocity and the risk of low bone mineral density (BMD) in children.

**Design:**

A population-based prospective cohort study.

**Patients and methods:**

A total of 10 450 participants with complete follow-up records from a cohort were included in the current study. Serum 25(OH)D concentrations were measured at baseline and 2-year follow-up, and the average of 2-time measurements was used for analysis. Low BMD was defined as calcaneus speed of sound Z-score ≤ −1. The associations of vitamin D with height growth velocity and the risks of incident low BMD were evaluated using adjusted β and risk ratio (*RR*).

**Results:**

After multivariable adjustment, an inverse L-shaped association between serum 25(OH)D concentrations and height growth velocity was observed, leveling off up to 40–60 nmol/L. Overall, each 10 nmol/L higher serum 25(OH)D concentration was associated with a 0.15 cm/year higher height growth velocity (*P* < 0.001) and a 7% decreased risk of low BMD [*RR* (95%*CI*): 0.93 (0.87~0.98)]. Compared to those with vitamin D deficiency, participants who had sufficient vitamin D had a 22% lower risk for low BMD [*RR*(95%*CI*): 0.78 (0.62~0.98)]. However, no significant associations between vitamin D and the risk of low BMD were found in overweight and obese children.

**Conclusion:**

These findings highlight the importance of maintenance of sufficient 25(OH)D concentrations and healthy body weight during childhood in height growth and bone health promotion.

## Introduction

Vitamin D is a fat-soluble micronutrient that can promote calcium absorption in the gut and maintain serum calcium and phosphorus homeostasis (1), and is thus needed for bone growth and bone remodeling by osteoblasts and osteoclasts (2). The deficiency of vitamin D is linked to pediatric rickets and adult osteomalacia (3). However, poor vitamin D status is reported worldwide and estimated to affect nearly 1 billion people with the largest prevalence in children (4).

Generally, vitamin D is considered to enhance childhood growth, although evidence is limited and conflicting. Several epidemiological studies have investigated the effects of maternal or infant vitamin D on height and bone growth (5–12). However, most of the studies were based on the populations below 6 years of age (5–7, 9–12), and some discrepancies existed in their findings due to a limited sample size (< 10,000) (7, 8, 10, 12). For instance, a randomized controlled trial of 2,079 low birthweight infants found that a weekly dose of vitamin D (35 μg/week) resulted in better vitamin D status and benefited weight and length growth at 6 months (12). In contrast, findings from a recent cohort study of 791 children aged 6–30 months were failed to support the viewpoint that poor vitamin D status in early childhood was an important limiting factor for linear growth (8). Additionally, the differences in the associations of vitamin D with height and bone development across gender, weight, and sexually mature status are not well-understood. Adipose tissue can decrease the bioavailability of vitamin D by excess storage of it, and consequently may be an effect modifier of vitamin D function (13). Likewise, the biochemical interaction between vitamin D and growth hormone has been explored by an increasing number of biological studies, which indicates the growth promotion effect of vitamin D may vary according to the stage of development (14).

Therefore, prospective high-quality evidence focusing on the relationships between vitamin D and height and bone growth in school-aged children are still needed to fill the knowledge gaps. In this large population-based cohort study from the School-based Cardiovascular and Bone Health Promotion Program (SCVBH), we aimed to investigate the associations of vitamin D with height growth velocity and low bone mineral density (BMD) in children aged 6~16 years, and further explore the effect modification of gender, age, obesity, and sexual maturity on these associations.

## Materials and methods

### Study population

The SCVBH program is an ongoing survey study of cardiovascular and bone health among school-aged children in Beijing (Latitude: 39°56′ N, Longitude: 116°20′ E), China. The study design in detail has been reported elsewhere (15, 16). In brief, survey of questionnaire, physical measurements, and laboratory assay was conducted in 2 waves. In the baseline wave during November to December 2017, a representative sample of 15 391 children was recruited from the general population using a stratified cluster sampling method. To achieve a high follow-up rate, we chose the students aged 6–16 years from Grade 1 to Grade 3, Junior 1 and Senior 1 as the target group. The second wave successfully followed up 12 984 participants between November to December 2019, with a follow-up rate of 84%.

**Figure 1:**
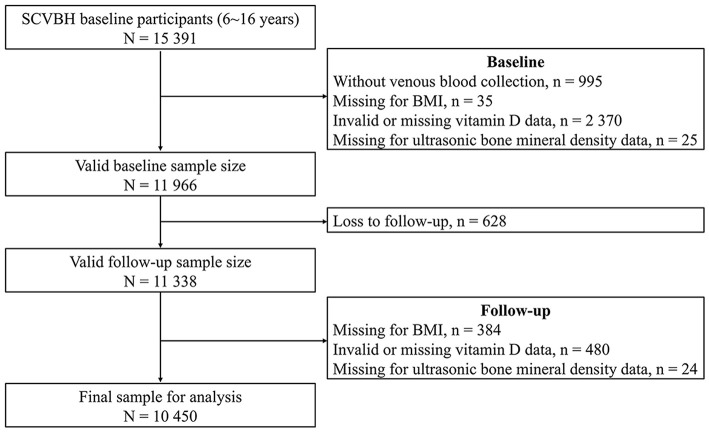
Flow diagram of study population selection.

In the current analysis, we included participants with valid serum 25-hydroxyvitamin D (25[OH]D) concentrations, body mass index (BMI), and bone mineral density (BMD) data at baseline (*n* = 11,966), and further excluded those who were lost to follow-up (*n* = 628) and had no available data on 25(OH)D, BMI, and BMD (*n* = 888) at follow-up. In total, 10 450 participants were included in the final analysis (Figure 1).

The SCVBH program received ethical approval from the Institutional Review Boards of Capital Institute of Pediatrics (approval number: SHERLL2016026), and all the participants aged ≥12 years and parents of participants aged < 12 years provided written informed consent.

### Ascertainment of covariates

Socio-demographic factors (age, sex, grade, and family income), lifestyle habits (smoking, drinking, physical activity, and dietary habits), oral calcium and vitamin D supplements, sexual maturity, and parents' height were collected by pretested and validated questionnaires. Smoking was defined as having at least one complete cigarette in the past month (17). Drinking behavior was classified as those who consumed more than a standard amount of alcoholic beverage (e.g., 120 ml wine, 50 ml liquor, or a bottle of beer) in the past month (18). Frequency (per week), duration (minutes), and intensity (vigorous or moderate) in a typical week were assessed for physical activity, and a daily frequency of moderate or vigorous physical activity ≥60 min was regarded as an ideal status (17). A validated food frequency questionnaire was used to collect the frequency of different food intakes in the past half-year. Dietary components related to both the vitamin D and height growth were adjusted in our analyses, including bean-curd or dairy products, aquatic foods, fruits and vegetables, meat products. And the dietary components were analyzed as binary variables according to whether the consumption of them ≥1 time/day (17, 19). We categorized annual household income into three groups: high (>250,000 yuan), medium (50,000 ~ 250,000 yuan), and low (< 50,000 yuan). The occurrence and date of first spermatorrhea for boys or menstruation for girls was collected by self-report, and then sexual maturity status was judged. The usage of vitamin D and calcium supplementation was collected by asking whether the participants had supplementation in the past 6 months. To better control the long-term effects of covariates on vitamin D levels and height growth, both the baseline and follow-up information on usage of vitamin D and calcium supplementation were adjusted in the multivariable analyses. Body mass index (BMI) was calculated as weight (kg) divided by height (m^2^). Weight status was categorized as normal, overweight, and obesity according to the sex- and age-specific BMI cutoff points for Chinese children (20). Serum calcium level was measured using Hitachi 7,080 automatic biochemical analyzer (Hitachi High-Technologies Corporation, Tokyo, Japan). Information on the birth status, including gestational week (term birth or not), birth weight, and infancy breastfeeding status (exclusive breastfeeding or not), were adjusted in multivariable analyses.

### Ascertainment of serum 25(OH)D concentrations and vitamin D status

Serum 25(OH)D concentrations were determined at both the baseline and follow-up using DiaSorin 25OH Vitamin D total assay (DiaSorin, Stillwater, MN, USA) on an automated chemiluminescent platform. To better approximate long-term vitamin D status, the mean of 25(OH)D concentrations at two times was used for analysis. Vitamin D status was classified as deficiency (< 30 nmol/L), insufficiency (30 ~ < 50 nmol/L), and sufficiency (≥ 50 nmol/L) according to the Institute of Medicine recommendations (2).

### Evaluation of height growth velocity and low bone mineral density

Height and BMD were measured both at the baseline and follow-up. Height growth velocity (cm/year) was assessed by the annual height increment, which was calculated by dividing the individuals' height difference (cm) between the baseline and follow-up by follow-up duration (year). The speed of sound (SOS) through the calcaneus was measured to assess the BMD using a quantitative ultrasound system (CM-200, Furuno Inc., Nishinomiya, JP). Sex- and age-specific *Z*-scores of the SOS were calculated, and low BMD was defined as *Z*-score ≤ −1 (21).

### Statistical analysis

Baseline characteristics were presented as mean (SD) for continuous variables and frequency (%) for categorical variables. Differences in characteristics across vitamin D status groups were tested with a one-way analysis of variance (continuous data) and Pearson χ^2^ test (categorical data). A generalized additive model with penalized spline terms was implemented to explore the dose-response associations of serum 25(OH)D concentrations with height growth velocity and the incidence of low BMD during the follow-up period. Multivariable linear regression analyses were used to investigate the association between serum 25(OH)D concentrations and height growth velocity. The estimated marginal mean of height growth velocity in each vitamin D status group was calculated using analysis of covariance, and the Tukey-Kramer test was used to compare the mean between each pairwise combination of groups in the *post-hoc* analysis. The cumulative incidence rate (*CIR*) of low BMD were calculated by dividing the number of new cases within a 2-year follow-up period by the number of participants who were free of the corresponding outcome at baseline. The relationships between serum 25(OH)D concentrations or vitamin D status and risk of low BMD were determined by risk ratios (*RRs*) derived from multivariable log-binomial regressions. The trends of risks across vitamin D status were examined by assigning numeric values (1, 2, and 3) to the variable in the models.

Stratified analyses were also conducted according to age, sex, weight status, and sexual maturity. To further examine whether the associations differed by these stratification variables, the potential effect modification was assessed using a two-way product term in the models. Sensitivity analyses were performed by transforming raw height growth velocity (cm/year) into sex- and age-specific *Z*-scores in the models. Statistical analyses were performed using *R* software (version 3.4.0, www.cran.r-project.org), and a two-tailed *P* ≤ 0.05 was considered statistically significant.

## Results

### Characteristics of study population

Among the 10 450 participants, the mean (SD) age was 10.9 (3.1) years, and 5 185 (49.6%) were boys. Overall, the mean (SD) of serum 25(OH)D concentration was 38.5 (11.7) nmol/L, and the prevalence of vitamin D deficiency, insufficiency, and sufficiency were 25.0, 59.4, and 15.6%, respectively. The mean (SD) height growth velocity for vitamin D deficiency, insufficiency, and sufficiency groups were 3.5 (3.1), 4.5 (2.9), and 5.2 (2.5) cm/year, respectively. The 2-year *CIR* of low BMD among the study population were 10.4%. The characteristics of the study population according to vitamin D status are shown in Table 1. Participants with higher serum vitamin D levels were more likely to be younger, boys, term birth, achieving ideal physical activity, having bean-curd or dairy products, and using vitamin D and calcium supplements (*P* < 0.05). Interestingly, compared to those with lower serum vitamin D levels, participants with higher levels of vitamin D had significantly lower levels of BMI, height, and calcaneal speed of sound at both the baseline and follow-up (*P* < 0.05).

**Table 1:** Characteristics of study population.

**Characteristics**	**Overall**	**Vitamin D status**			
		**Deficiency** **(**<**30 nmol/L)**	**Insufficiency** **(30**~ < **50 nmol/L)**	**Sufficiency** **(**≥**50 nmol/L)**	***P*** **-value**
*n*	10,450	2,611	6,205	1,634	-
Age, mean (SD), year	10.9 (3.1)	12.0 (3.1)	10.8 (3.3)	9.8 (3.3)	< 0.001
Boys, *n* (%)	5,185 (49.6)	903 (34.6)	3,188 (51.4)	1,094 (67.0)	< 0.001
Smoking, *n* (%)	119 (1.1)	20 (0.8)	82 (1.3)	17 (1.0)	0.074
Drinking, *n* (%)	692 (6.6)	215 (8.2)	380 (6.1)	97 (5.9)	0.001
Bean-curd or dairy products ≥1 time/day, *n* (%)	4,051 (38.8)	928 (35.5)	2,414 (38.9)	709 (43.4)	< 0.001
Aquatic foods ≥1 time/day, *n* (%)	214 (2.0)	52 (2.0)	126 (2.0)	36 (2.2)	0.884
Fruits and vegetables ≥1 time/day, *n* (%)	5,504 (52.7)	1,379 (52.8)	3,291 (53.0)	834 (51.0)	0.350
Meat products ≥1 time/day, *n* (%)	3,833 (36.7)	957 (36.7)	2,258 (36.4)	618 (37.8)	0.565
Term birth, *n* (%)	8,292 (79.3)	2,027 (77.6)	4,937 (79.6)	1,328 (81.3)	0.014
Birth weight, kg	3.4 (0.6)	3.4 (0.6)	3.4 (0.6)	3.4 (0.6)	0.252
Exclusive breastfeeding, *n* (%)	4,100 (39.2)	1,064 (40.8)	2,431 (39.2)	605 (37.0)	0.053
Ideal physical activity, *n* (%)	567 (5.4)	127 (4.9)	320 (5.2)	120 (7.3)	0.001
Sexual maturity, *n* (%)	3,021 (28.9)	1,126 (43.1)	1,671 (26.9)	224 (13.7)	< 0.001
Annual household income, *n* (%)					0.215
High	1,671 (16.0)	425 (16.3)	993 (16.0)	253 (15.5)	
Medium	6,834 (65.4)	1,678 (64.3)	4,051 (65.3)	1,105 (67.6)	
Low	1,945 (18.6)	508 (19.5)	1,161 (18.7)	276 (16.9)	
Father height, mean (SD), cm	173.9 (5.2)	173.7 (5.5)	174.0 (5.1)	174.0 (5.3)	0.046
Mother height, mean (SD), cm	161.9 (4.9)	161.8 (5.2)	161.9 (4.9)	162.0 (4.7)	0.307
**Baseline characteristics**					
Usage of vitamin D supplements, *n* (%)	2,077 (19.9)	433 (16.6)	1,233 (19.9)	411 (25.2)	< 0.001
Usage of calcium supplements, *n* (%)	3,076 (29.4)	661 (28.2)	1,837 (32.8)	578 (39.3)	< 0.001
Serum calcium, mean (SD), mmol/L	2.46 (0.11)	2.45 (0.10)	2.45 (0.11)	2.47 (0.11)	< 0.001
BMI, mean (SD), kg/m^2^	20.2 (4.6)	20.7 (4.7)	20.2 (4.7)	19.5 (4.4)	< 0.001
Weight status					< 0.001
Normal	6,185 (58.9)	1,649 (63.2)	3,565 (57.5)	944 (57.8)	
Overweight	1,865 (17.8)	401 (15.4)	1,152 (18.6)	312 (19.1)	
Obesity	2,427 (23.2)	561 (21.5)	1,488 (24.0)	378 (23.1)	
Height, mean (SD), cm	149.0 (17.9)	153.5 (15.6)	148.5 (18.0)	143.8 (19.0)	< 0.001
Calcaneal speed of sound, mean (SD), m/s	1,538.6 (88.8)	1,540.1 (41.6)	1,539.1 (109.5)	1,534.3 (46.8)	0.095
**Follow-up characteristics**					
Usage of vitamin D supplements, *n* (%)	2,077 (19.9)	433 (16.6)	1,233 (19.9)	411 (25.2)	< 0.001
Usage of calcium supplements, *n* (%)	3,086 (29.5)	600 (23.0)	1,881 (30.3)	605 (37.0)	< 0.001
Serum calcium, mean (SD), mmol/L	2.48 (0.13)	2.46 (0.13)	2.48 (0.13)	2.51 (0.14)	< 0.001
BMI, mean (SD), kg/m^2^	21.3 (4.9)	21.8 (5.0)	21.3 (4.92)	20.5 (4.6)	< 0.001
Weight status					< 0.001
Normal	6,250 (59.8)	1,648 (63.1)	3,613 (58.2)	989 (60.5)	
Overweight	1,932 (18.5)	429 (16.4)	1,201 (19.4)	302 (18.5)	
Obesity	2,268 (21.7)	534 (20.5)	1,391 (22.4)	343 (21.0)	
Height, mean (SD), cm	157.7 (14.5)	160.5 (11.9)	157.4 (14.7)	154.1 (16.3)	< 0.001
Calcaneal speed of sound, mean (SD), m/s	1,541.4 (33.5)	1,545.2 (32.2)	1,540.6 (31.9)	1,538.3 (40.1)	< 0.001

SD, standard deviation; BMI, body mass index.

### Associations of vitamin D with height growth velocity

**Figure 2:**
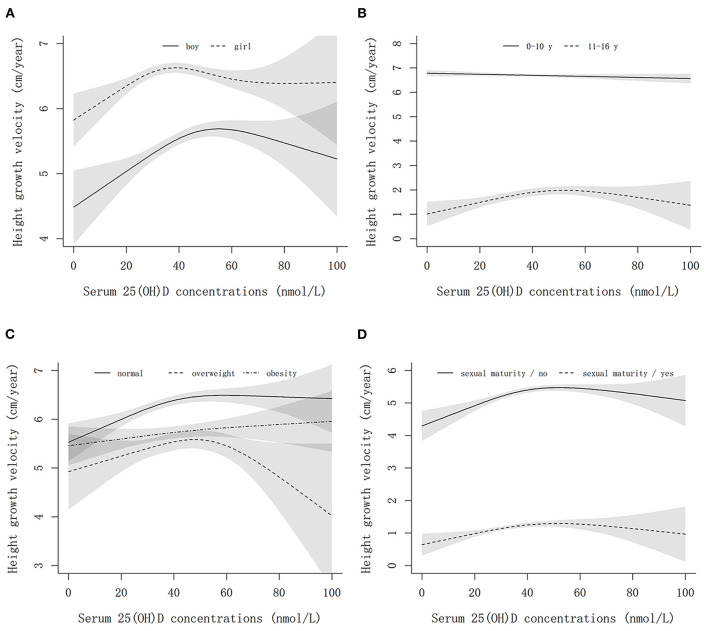
The non-linear associations between serum 25(OH)D concentrations and height growth velocity by sex, age, weight, and sexual maturity status. **(A–D)** Show the results stratified by sex, age, weight status, and sexual maturity status, respectively. The models are adjusted for age, sex (except for stratified analysis), family income, smoking, drinking, bean-curd or dairy products, aquatic foods, fruits and vegetables, meat products, term birth, birth weight, exclusive breastfeeding, usage of vitamin D / calcium supplements in baseline and follow-up, physical activity, serum calcium, BMI, parents' height, sexual maturity (except for stratified analysis), and baseline height.

**Figure 3:**
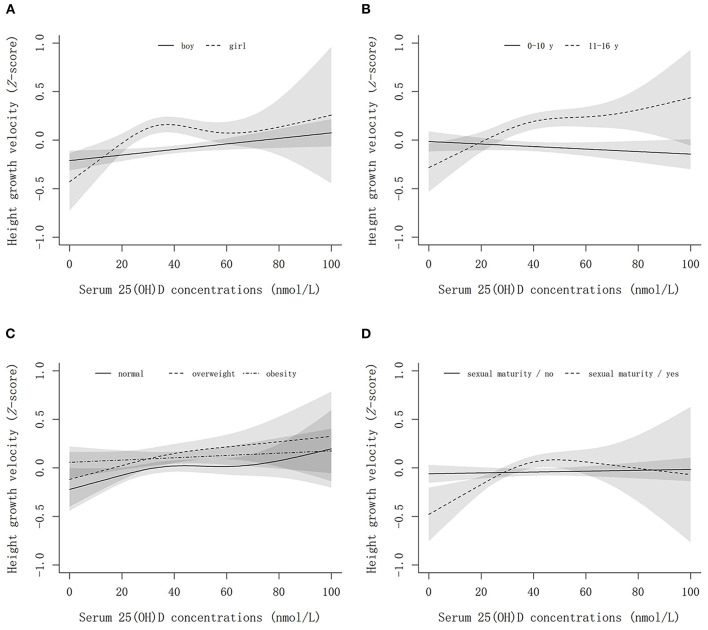
The non-linear associations between serum 25(OH)D concentrations and height growth velocity *Z*-score by sex, age weight, and sexual maturity status. **(A–D)** Show the results stratified by sex, age, weight status, and sexual maturity status, respectively. The models are adjusted for age, sex (except for stratified analysis), family income, smoking, drinking, bean-curd or dairy products, aquatic foods, fruits and vegetables, meat products, term birth, birth weight, exclusive breastfeeding, usage of vitamin D / calcium supplements in baseline and follow-up, physical activity, serum calcium, BMI, parents' height, sexual maturity (except for stratified analysis), and baseline height.

Figure 2 shows the non-linear associations between serum 25(OH)D concentrations and height growth velocity by sex, age, weight, and sexual maturity status. Elevated serum 25(OH)D concentrations were positively associated with height growth velocity after multivariable adjustment, and an inverse L-shaped association between them was observed, leveling off up to 40–60 nmol/L. Interestingly, we found that the optimum vitamin D level for boys to promote height growth was ~50 nmol/L, while that for girls was 40 nmol/L (Figure 2A). In the sensitivity analysis, an approximately positive linear association between serum 25(OH)D concentrations and age- and sex-specific *Z*-score of height growth velocity was found (Figure 3).

**Figure 4:**
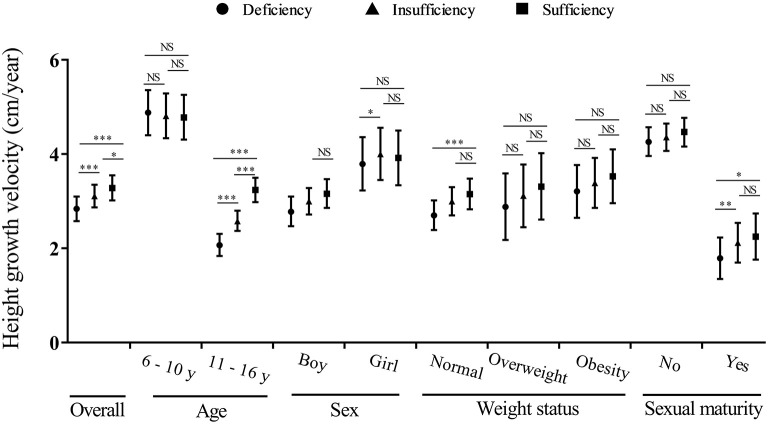
The estimated marginal mean of height growth velocity in different vitamin D status groups by sex, age, weight, and sexual maturity status. *0.01 ≤ *P* < 0.05; **0.001 ≤ *P* < 0.01; ***P < 0.001; NS, not significant. The models are adjusted for age, sex (except for stratified analysis), family income, smoking, drinking, bean-curd or dairy products, aquatic foods, fruits and vegetables, meat products, term birth, birth weight, exclusive breastfeeding, usage of vitamin D / calcium supplements in baseline and follow-up, physical activity, serum calcium, BMI, parents' height, sexual maturity (except for stratified analysis), and baseline height.

Overall, each 10 nmol/L higher serum 25(OH)D concentration was associated with a 0.15 cm/year higher height growth velocity (Table 2). And no significant differences in the associations between continuous 25(OH)D concentrations and height growth velocity were found across different sex and weight status groups (*P*_interaction_ > 0.05). However, the linear associations between serum 25(OH)D concentration and height growth velocity differed according to age and sexual maturity status (*P*_interaction_ < 0.05). Estimated marginal means of height growth velocity in each vitamin D status group are given in Table 2, and Figure 4 shows the *post-hoc* analysis of pairwise mean comparison. In boys, the vitamin D sufficiency group had the highest mean of height growth velocity, which was significantly higher than the vitamin D deficiency group (3.17 vs. 2.77 cm/year, *P* = 0.003). Among girls, the mean height growth velocity was highest in the vitamin D insufficiency group, which showed significantly higher (4.01 vs. 3.78 cm/year, *P* = 0.011) than that in the vitamin D deficiency group but no significant difference (4.01 vs. 3.93 cm/year, *P* = 0.703) with that in the vitamin D sufficiency group. Notably, in normal-weight children, both the mean height growth velocity in the vitamin D sufficiency and insufficiency group were significantly higher (*P* < 0.001) than that in the vitamin D deficiency group, while no significant differences were found across vitamin D status groups in overweight and obese children.

**Table 2:** Associations between serum 25(OH)D and height growth velocity (cm/year).

**Subgroup**	**Continuous 25(OH)D**				**Vitamin D status**						
	**concentrations per 10 nmol/L**										
					**Deficiency**		**Insufficiency**		**Sufficiency**		*P*_trend_
	β	**SE**	***P*** **-value**	*P*_interaction_	**Mean (95%** ***CI*** **)**	**SE**	**Mean (95%** ***CI*** **)**	**SE**	**Mean (95%** ***CI*** **)**	**SE**	
Overall	0.15	0.025	< 0.001		2.83 (2.57~3.09)	0.135	3.13 (2.90~3.38)	0.135	3.30 (3.04~3.57)	0.145	< 0.001
Sex				0.786							
Boy	0.12	0.035	< 0.001		2.77 (2.46~3.09)	0.163	3.01 (2.73~3.29)	0.145	3.17 (2.87~3.48)	0.157	0.001
Girl	0.07	0.032	0.085		3.78 (3.22~4.35)	0.289	4.01 (3.46~4.57)	0.287	3.93 (3.35~4.51)	0.297	0.055
Age				< 0.001							
6–10 y	−0.01	0.020	0.268		4.89 (4.41~5.37)	0.245	4.82 (4.35~5.30)	0.242	4.79 (4.32~5.27)	0.249	0.133
11–16 y	0.05	0.022	0.007		2.06 (1.83~2.30)	0.120	2.57 (2.36~2.79)	0.111	3.25 (2.99~3.51)	0.135	< 0.001
Weight status				0.098							
Normal	0.14	0.025	< 0.001		2.71 (2.40~3.03)	0.163	3.01 (2.71~3.31)	0.163	3.17 (2.85~3.50)	0.169	< 0.001
Overweight	0.10	0.066	0.103		2.89 (2.19~3.60)	0.365	3.11 (2.44~3.77)	0.342	3.32 (2.61~4.02)	0.362	0.046
Obesity	0.09	0.058	0.051		3.20 (2.64~3.76)	0.286	3.40 (2.87~3.93)	0.273	3.54 (2.96~4.10)	0.294	0.069
Sexual maturity				< 0.001							
No	0.08	0.029	0.003		4.27 (3.97~4.58)	0.159	4.37 (4.08~4.66)	0.149	4.48 (4.17~4.78)	0.158	0.029
Yes	−0.02	0.033	0.702		1.80 (1.36~2.24)	0.235	2.13 (1.71~2.55)	0.215	2.26 (1.77~2.75)	0.252	< 0.001

CI, confidence interval. SE, standard error. β was calculated by using multivariable linear regression and the estimated marginal mean of height growth velocity in each group was calculated by using Analysis of Covariance. The models are adjusted for age, sex (except for stratified analysis), family income, smoking, drinking, bean-curd or dairy products, aquatic foods, fruits and vegetables, meat products, term birth, birth weight, exclusive breastfeeding, usage of vitamin D / calcium supplements in baseline and follow-up, physical activity, serum calcium, parents' height, sexual maturity (except for stratified analysis), and baseline height.

**Figure 5:**
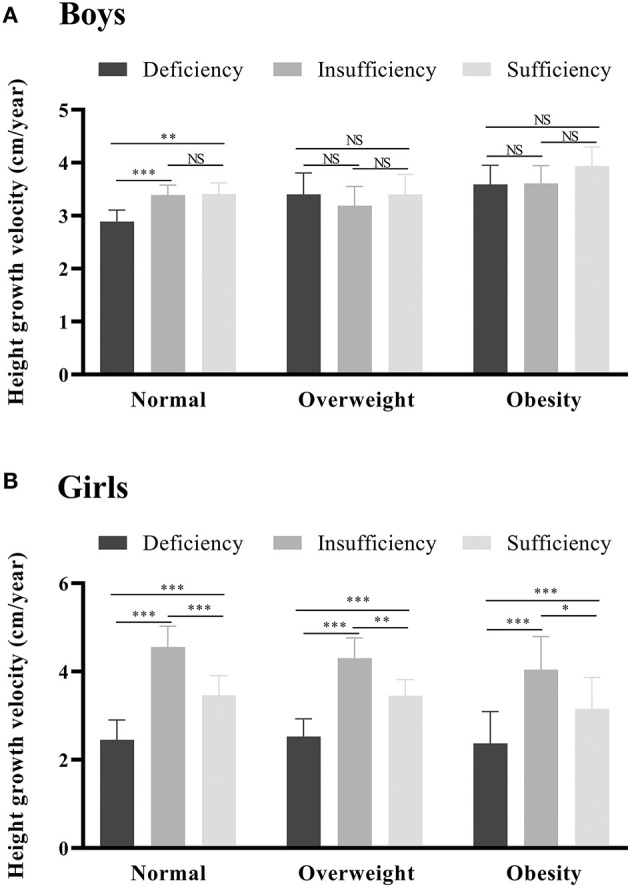
The estimated marginal mean of height growth velocity in different vitamin D status groups by sex across the weight status. *0.01 ≤ *P* < 0.05; **0.001 ≤ *P* < 0.01; ***P < 0.001; NS, not significant. **(A, B)** stand for boys and girls, respectively. The models are adjusted for age, family income, smoking, drinking, bean-curd or dairy products, aquatic foods, fruits and vegetables, meat products, term birth, birth weight, exclusive breastfeeding, usage of vitamin D / calcium supplements in baseline and follow-up, physical activity, serum calcium, BMI, parents' height, sexual maturity (except for stratified analysis), and baseline height.

In addition, estimated marginal means of height growth velocity according to sex and weight status were calculated to evaluate the influence of combined factors (Table 3). For boys, the obese and vitamin D sufficiency group had the highest height growth velocity (3.94, 95%*CI*: 3.24~4.64). Nevertheless, the highest height growth velocity (4.56, 95%*CI*: 3.64~5.47) came from the normal weight and vitamin D insufficiency group in girls. The *post-hoc* analysis of pairwise mean comparison found that the height growth velocity was not significant different across vitamin D status groups in overweight and obese boys (Figure 5A). However, for girls, the height growth velocity was significantly higher in vitamin D insufficiency group compared to the deficiency and sufficiency group in either weight status group (Figure 5B).

**Table 3:** Associations between serum 25(OH)D and height growth velocity (cm/year) by sex across the weight status.

**Subgroup**	**Continuous 25(OH)D**				**Vitamin D status**						
	**concentrations per 10 nmol/L**				**Deficiency**		**Insufficiency**		**Sufficiency**		*P*_trend_
	β	**SE**	***P*** **-value**	*P*_interaction_	**Mean (95%** ***CI*** **)**	**SE**	**Mean (95%** ***CI*** **)**	**SE**	**Mean (95%** ***CI*** **)**	**SE**	
Boys				0.206							
Normal	0.15	0.041	< 0.001		2.89 (2.46~3.31)	0.217	3.39 (3.02~3.76)	0.189	3.41 (3.00~3.82)	0.209	0.003
Overweight	0.01	0.069	0.856		3.40 (2.60~4.19)	0.406	3.19 (2.48~3.90)	0.361	3.40 (2.65~4.15)	0.383	0.827
Obesity	0.09	0.059	0.147		3.59 (2.88~4.30)	0.363	3.61 (2.96~4.26)	0.332	3.94 (3.24~4.64)	0.356	0.120
Girls				0.334							
Normal	0.04	0.037	0.321		2.45 (1.57~3.34)	0.453	4.56 (3.64~5.47)	0.466	3.46 (2.58~4.33)	0.448	0.321
Overweight	0.09	0.093	0.336		2.53 (1.74~3.31)	0.398	4.30 (3.39~5.21)	0.462	3.45 (2.74~4.17)	0.365	0.225
Obesity	0.07	0.082	0.399		2.37 (0.95~3.78)	0.720	4.04 (2.56~5.52)	0.754	3.15 (1.74~4.55)	0.716	0.143

CI, confidence interval. SE, standard error. β was calculated by using multivariable linear regression and the estimated marginal mean of height growth velocity in each group was calculated by using Analysis of Covariance. The models are adjusted for age, sex (except for stratified analysis), family income, smoking, drinking, bean-curd or dairy products, aquatic foods, fruits and vegetables, meat products, term birth, birth weight, exclusive breastfeeding, usage of vitamin D / calcium supplements in baseline and follow-up, physical activity, serum calcium, parents' height, sexual maturity (except for stratified analysis), and baseline height.

### Associations of vitamin D with low bone mineral density

**Figure 6:**
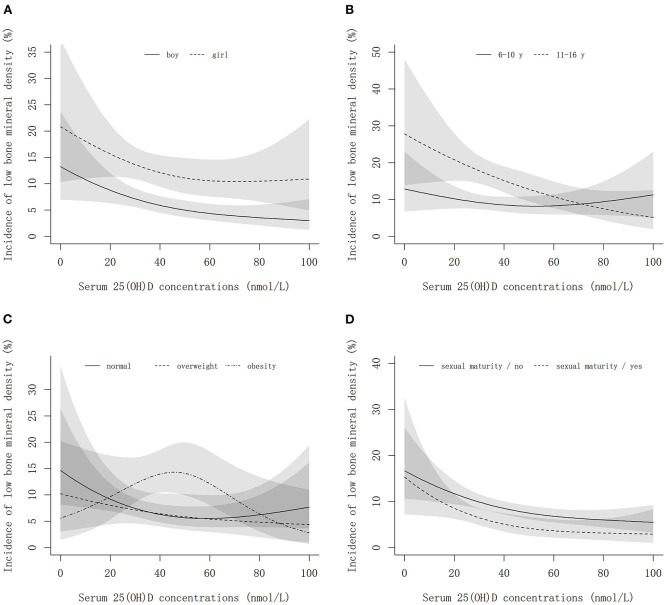
The non-linear associations of serum 25(OH)D concentrations with the incidence of low bone mineral density by sex, age, weight, and sexual maturity status. **(A–D)** Show the results stratified by sex, age, weight status, and sexual maturity status, respectively. The models are adjusted for age, sex (except for stratified analysis), family income, smoking, drinking, bean-curd or dairy products, aquatic foods, fruits and vegetables, meat products, term birth, birth weight, exclusive breastfeeding, usage of vitamin D / calcium supplements in baseline and follow-up, physical activity, serum calcium, BMI, parents' height, sexual maturity (except for stratified analysis), and baseline calcaneal speed of sound.

Table 4 shows that the estimated marginal means of calcaneus speed of sound (*Z*-score) at follow-up increased with higher vitamin D levels (*P*_trend_ < 0.001). The best bone mineral density was achieved in vitamin D sufficiency group in both sexes. The incidence of low BMD decreased curvilinearly with the increment of serum 25(OH)D concentrations in the study population, except for an inverse U-shaped association observed in obese children (Figure 6). Table 5 shows the associations of low BMD risk with continuous 25(OH)D concentrations and categorical vitamin D status. Overall, each 10 nmol/L higher serum 25(OH)D concentration was associated with about a 7% (95%*CI*: 2~13%) decreased risk of low BMD. The stratified analyses found that each 10 nmol/L higher serum 25(OH)D concentration was associated with about 9, 8, and 7% decreased risk of low BMD in boys, normal weight, and sexual immature children, respectively, but no significant associations between them were found in girls, overweight, obese, and sexual mature children. Compared to those with vitamin D deficiency, participants who had sufficient vitamin D levels had a significantly decreased risk of low BMD in boys and normal-weight children. However, in overweight and obese children, no significant differences in the risk of low BMD were found across different vitamin D status groups.

**Table 4:** The estimated marginal means of calcaneus speed of sound (*Z*-score) at follow-up by sex across the weight status^a^.

**Subgroup**	**Vitamin D status**						
	**Deficiency**		**Insufficiency**		**Sufficiency**		*P*_trend_
	**Mean (95%** ***CI*** **)**	**SE**	**Mean (95%** ***CI*** **)**	**SE**	**Mean (95%** ***CI*** **)**	**SE**	
**Boys**							
Normal	0.03 (−0.14~0.19)	0.08	0.14 (0.01~0.29)	0.07	0.30 (0.14~0.46)	0.08	< 0.001
Overweight	−0.19 (−0.51~0.13)	0.16	0.01 (−0.28~0.30)	0.14	0.10 (−0.21~0.40)	0.15	< 0.001
Obesity	−0.17 (−0.43~0.10)	0.13	−0.04 (−0.29~0.20)	0.12	0.12 (−0.15~0.38)	0.13	< 0.001
**Girls**							
Normal	0.13 (−0.16~0.42)	0.15	0.28 (−0.01~0.57)	0.15	0.31 (0.01~0.61)	0.15	< 0.001
Overweight	0.05 (−0.21~0.30)	0.13	0.18 (−0.05~0.41)	0.12	0.36 (0.07~0.65)	0.15	< 0.001
Obesity	−0.08 (−0.63~0.47)	0.09	0.02 (−0.52~0.57)	0.15	0.41 (−0.16~0.99)	0.13	< 0.001

a 1,780 participants with low bone mineral density at baseline were excluded, and the analytic sample size was 8,670. CI, confidence interval. SE, standard error. The estimated marginal mean of height growth velocity in each group was calculated by using Analysis of Covariance. The models are adjusted for age, sex (except for stratified analysis), family income, smoking, drinking, bean-curd or dairy products, aquatic foods, fruits and vegetables, meat products, term birth, birth weight, exclusive breastfeeding, usage of vitamin D / calcium supplements in baseline and follow-up, physical activity, serum calcium, parents' height, sexual maturity (except for stratified analysis), and baseline calcaneal speed of sound.

**Table 5:** Associations between serum 25(OH)D and incident low bone mineral density^a^.

**Subgroup**	**Continuous 25(OH)D**		**Vitamin D status**						
	**concentrations per 10 nmol/L**								
			**Deficiency**		**Insufficiency**		**Sufficiency**		*P*_trend_
	***RR*** **(95%*****CI*****)**	*P*_interaction_	***CIR*** **(95%*****CI*****)**	***RR***	***CIR*** **(95%*****CI*****)**	***RR*** **(95%*****CI*****)**	***CIR*** **(95%*****CI*****)**	***RR*** **(95%*****CI*****)**	
Overall	0.93 (0.87~0.98)^*^		10.3 (9.0~11.7)	Reference	10.4 (9.5~11.2)	0.83 (0.72~0.96)^*^	10.9 (9.3~12.7)	0.78 (0.62~0.98)^*^	0.013
Sex		0.116							
Boy	0.91 (0.84~0.99)^*^		11.7 (9.4~14.2)	Reference	10.8 (9.7~12.1)	0.80 (0.62~1.03)	9.8 (8.0~11.9)	0.62 (0.45~0.87)^**^	0.005
Girl	0.95 (0.86~1.05)		9.5 (8.0~11.2)	Reference	9.8 (8.7~11.1)	0.81 (0.65~1.02)	12.1 (9.3~15.4)	0.82 (0.59~1.16)	0.130
Age		0.839							
6–10 y	0.97 (0.89~1.06)		11.8 (9.6~14.2)	Reference	10.4 (9.3~11.6)	0.91 (0.71~1.16)	10.9 (8.9~13.2)	0.98 (0.72~1.34)	0.879
11–16 y	0.82 (0.74~0.90)^***^		9.3 (7.8~11.1)	Reference	10.2 (9.1~11.5)	0.76 (0.60~0.96)^*^	10.0 (7.5~12.9)	0.54 (0.37~0.77)^***^	< 0.001
Weight status		0.998							
Normal	0.92 (0.85~0.99)^*^		11.3 (9.6~13.1)	Reference	9.9 (8.9~11.0)	0.70 (0.57~0.87)^**^	11.7 (9.6~14.1)	0.72 (0.53~0.97)^*^	0.022
Overweight	0.93 (0.80~1.08)		9.0 (6.1~12.8)	Reference	10.8 (8.9~13.0)	0.97 (0.62~1.55)	9.8 (6.5~14.0)	0.76 (0.4.~1.35)	0.428
Obesity	0.88 (0.79~1.04)		8.3 (5.8~11.2)	Reference	11.1 (9.4~13.0)	1.11 (0.75~1.63)	8.3 (5.5~12.1)	0.73 (0.41~1.25)	0.371
Sexual maturity		0.378							
No	0.93 (0.86~0.99)^*^		11.6 (9.9~13.5)	Reference	11.1 (10.1~12.1)	0.86 (0.71~1.05)	10.9 (9.2~12.8)	0.79 (0.61~0.99)^*^	0.058
Yes	0.87 (0.74~1.02)		8.3 (6.5~10.4)	Reference	8.3 (6.9~9.9)	0.75 (0.55~1.03)	8.7 (5.2~13.6)	0.56 (0.32~0.99)^*^	0.035

RR, risk ratio. CI, confidence interval. CIR, cumulative incidence rate.

a 1,780 participants with low bone mineral density at baseline were excluded, and the analytic sample size was 8,670.

* 0.01 ≤ P < 0.05;

** 0.001 ≤ P < 0.01;

*** P ≤ 0.001.

The models are adjusted for age, sex (except for stratified analysis), family income, smoking, drinking, bean-curd or dairy products, aquatic foods, fruits and vegetables, meat products, term birth, birth weight, exclusive breastfeeding, usage of vitamin D / calcium supplements in baseline and follow-up, physical activity, serum calcium, parents' height, sexual maturity (except for stratified analysis), and baseline calcaneal speed of sound.

## Discussion

Using this large population-based prospective cohort, we found that higher serum 25(OH)D concentrations in children were associated with increased height growth velocity and reduced risk of incident low BMD outcome. Furthermore, the dose-response analyses found the optimum vitamin D levels for height growth were ~50 and 40 nmol/L in boys and girls, respectively. In addition, for overweight and obese children, the height growth velocity and risk of low BMD did not differ significantly across vitamin D status groups.

Height growth is a complex physiological process that depends on the combined action of genetic, environmental, and nutritional factors (14). Vitamin D is an essential nutrient for calcium and bone homeostasis and, consequently, potentially has a promoted effect on height growth. However, the association between vitamin D and height growth has been evaluated in a limited number of studies, and few of them focused on the school-aged (>6 years) children (6, 7, 9, 11, 12, 22). To our knowledge, this is the first large population-based prospective analysis to investigate the associations between vitamin D and height growth velocity in children aged 6~17 years. A Cochrane review has found that, compared to the placebo or no intervention group, vitamin D supplementation (daily dose 200–2,000 IU) in children aged under 5 years old made little to no difference in height growth (mean difference: 0.66, 95%*CI*: −0.37~1.68 cm) (7). Similarly, a cohort study of 791 North Indian children aged 6~9 years did not support the notion that poor vitamin D status was a limiting factor for height growth (measured by height for age z-score) in early childhood [β(95%*CI*): −0.06(−0.24~0.11)] (8). In contrast, our current study found that per 10 nmol/L higher serum 25(OH)D concentration was associated with a 0.15 cm/year higher height growth velocity and 7% decreased risk of low BMD in children aged 6~17 years after adjusting for vitamin D / calcium supplements. And children with sufficient vitamin D levels had significantly higher height growth velocity than those with deficient vitamin D levels. The divergence of results across studies may attribute to the small sample size, different assessments of height growth, different classification methods of vitamin D levels, and inadequate adjustment of residual confounding. A *post-hoc* analysis of randomized controlled trial from Uday et al. highlighted the importance of overall calorie intake for the skeletal maturation in malnourished children (5). In their findings, quarterly oral vitamin D supplementation did not influence bone age delay, but total calorie intake did. Unlike other studies with one measurement of 25(OH)D concentrations, we used the mean of baseline and follow-up 25(OH)D concentrations for analyses, which might provide a more accurate estimation of vitamin D status during the period of follow-up. In addition, we further adjusted baseline height, birth weight and feeding, parents' height, and the usage of vitamin D / calcium supplements which were potential confounding factors. Moreover, our dose-response relationship analysis showed that an inverse L-shaped association between vitamin D and height growth velocity was observed, and thus this non-linear characteristic may lead to negative results when using linear statistical methods to analyze their relationships. Partially in line with our findings, a randomized clinical trial-based cohort in Northern Europe revealed that vitamin D had an inverse U-shaped association with early growth during the first 2 years of life (6). Therefore, a very high level of 25(OH)D concentration may have no or unfavorable effects on normal growth. Even though vitamin D has many important health benefits, there is controversy regarding how vitamin D deficiency should be defined and what requirements should be met (23). In the current study, the optimum vitamin D levels for height growth promotion in boys and girls were ~50 and 40 nmol/L, respectively. Thus, the highest growth rate came from vitamin D insufficiency group in girls, while that came from sufficiency group in boys. The difference in the optimum vitamin D levels between sexes may be partly explained by the sex dimorphism in body fat mass. Generally, girls have a significantly higher amount of adipose tissue deposition than boys (24), and the bioavailability of vitamin D may be decreased due to excess storage in body fat compartments (25). Thus, girls with sufficiency vitamin D levels did not achieve better height growth velocity as that in boys. This hypothesis was also supported by our results of non-significant associations between the vitamin D status and height growth velocity in obese children. Beyond the aforementioned mechanism, sexual hormones may also play an important role in the discrepancy of optimum levels of vitamin D between sexes. For example, testosterone synthesis in men is appeared to be affected by 25(OH)D (26), we assume that it is possible that higher 25(OH)D concentrations may improve testosterone levels and then increase the height growth velocity in boys. Our multiple linear analyses didn't find significant interactions between weight status and vitamin D on height growth in both sexes (Table 3). This may be due to some reasons such as non-linear association, sample size, residual confounding, etc. However, in contrast with higher height growth velocity observed in normal weight boys with higher vitamin D levels, the height growth velocity didn't significantly differ from vitamin D status in overweight and obese boys. We deduced this result may be partly explained by that the bioavailability of vitamin D decreased due to excess storage in body fat compartments (25). In other words, vitamin D facilitated height growth in normal weight boys. Still, the facilitation effect of vitamin D was no longer statistically significant in overweight and obese boys because the excess storage in body fat compartments decreased the bioavailability of vitamin D.

Beyond the effect of vitamin D on the maintenance of calcium and bone homeostasis, which is potentially the major mechanism underlying that vitamin D and height growth are intertwined, the relationship between vitamin D and growth hormone may also play an important role (14). Their linkage derives partly from the similarity of seasonal variability in the levels: vitamin D levels and stature growth are greater in the summer period, while they are lower during winter (27, 28). Our results revealed a significant interaction effect between vitamin D and sexual maturity status on height growth velocity. The biological mechanisms underpinning the interaction are not clear, but clues may be gained from other studies. Insulin-like growth factor 1 (IGF-1), the mediator of growth hormone (GH), has been found to stimulate the activity of the 1α-hydroxylase enzyme that, in turn, regulates the renal production of vitamin D (29). In addition, both GH and IGF-1 appear to increase CYP27A1 activity and, thus affect catalyzing the 25-hydroxylation of vitamin D (30). On the other hand, vitamin D appears to influence the hepatic secretion of IGF-1 and the expression of IGF-1 receptors (31).

Our current study also supported that higher serum 25(OH)D concentrations were associated with the decreased risk of low BMD in school-aged children. Consistent with our findings, the D-pro randomized trial, which evaluated the effects of vitamin D and high dairy protein intake on bone mineralization and linear growth, found that vitamin D could increase bone mass and spinal BMD in 6–8-year-old children (32). However, it is noteworthy that differing from the normal-weight children, the risk of low BMD does not vary with the changes in serum 25(OH)D concentrations among overweight and obese children in our study. We speculate that the explanation for this is the decreased bioavailability of vitamin D due to excess storage in body fat compartments (25). There is also evidence that the metabolic clearance of vitamin D may increase in obesity due to increased uptake by adipose tissue (33). We found that the vitamin D insufficiency girls achieved the best height growth velocity while those with sufficient vitamin D had best bone density. This results indicate that natural optimum vitamin D levels may vary according to age, sex, race, and health outcome et al., thus using a universal guideline for diagnosing deficiency may be inaccurate. It is now generally accepted that vitamin D deficiency is a worldwide health problem that affects not only musculoskeletal health but also a wide range of acute and chronic diseases. However, there remains cynicism about the lack of randomized controlled trials to support the association studies regarding the no skeletal health benefits of vitamin D. The role of vitamin D in no skeletal health has been under intense debate, with inconsistent results regarding its potential prognostic value and therapeutic role.

This study has several strengths. First, the SCVBH program is a well-established population-based prospective cohort with a large sample of school-aged children, which can avoid reverse causality in causal inference. Second, the dose-response analysis and adjustment of a multitude of potential confounding factors may enhance the validity of the conclusions. Finally, a Vitamin D Standardization-Certification Program certified assay was used to measure serum 25(OH)D concentrations at baseline and follow-up, and the 2-time points measurements of vitamin D were used for analysis. Some potential limitations should be also noted in the current study. First, the generalizability of the conclusion may be limited due to a lack of other ethnic groups in our study population. Second, residual confounding resulting from errors in the measurement of covariates and other unmeasured factors such as vitamin D fortified foods, sun exposure, cannot be eliminated. Finally, considering the large study sample size, we used an ultrasound bone densitometer to assess BMD and, thus may have a disparity in the accuracy.

## Conclusion

In conclusion, our results indicate that higher serum 25(OH)D levels were significantly associated with increased height growth velocity and decreased risk of low BMD in children aged 6~17 years. Furthermore, these associations were attenuated or eliminated in overweight and obese children. These findings highlight the importance of maintenance of sufficient 25(OH)D concentrations and healthy body weight during childhood in height growth and bone health promotion.

## Data availability statement

The raw data supporting the conclusions of this article will be made available by the authors, without undue reservation.

## Ethics statement

The studies involving human participants were reviewed and approved by Institutional Review Boards of Capital Institute of Pediatrics. Written informed consent to participate in this study was provided by the participants' legal guardian/next of kin.

## Author contributions

JM: conceptualization, methodology, supervision, and writing—original draft. PX and HC: data curation, formal analysis, and writing—original draft preparation. PX and HL: visualization and software. LW, DH, and XZ: investigation, reviewing, and editing. XX: methodology, supervision, and investigation. All authors contributed to the article and approved the submitted version.
